# Effect of external physical vibration lithecbole in obese patients with lower pole stones <15 mm after ESWL: a single-centre, randomized, open label clinical trial

**DOI:** 10.3389/fmed.2023.1101811

**Published:** 2023-08-30

**Authors:** Yunpeng Li, Jianlin Lv

**Affiliations:** Department of Urology, The Affiliated Jiangning Hospital of Nanjing Medical University, Nanjing, China

**Keywords:** external physical vibration lithecbole, extracorporeal shock wave lithotripsy, lower pole stones, stone-free rate, obese

## Abstract

**Objective:**

To investigate the efficacy and safety of external physical vibration lithecbole (EPVL) in obese patients with <15 mm lower pole stones following extracorporeal shock wave lithotripsy (ESWL).

**Methods:**

Two hundred and ninety-nine obese patients with BMI greater than 30 kg/m^2^ and lower pole stones smaller than 15 mm were prospectively randomized into two groups. While ESWL was the only option in the control group, patients in the treatment group accepted EPVL after receiving ESWL. Imaging tests were used to compare the stone expulsion status on day 1 and the stone-free rates (SFR) on the first, second, and fourth weekends.

**Results:**

All 299 obese patients were randomly divided into two groups, with 152 patients assigned to the treatment group and 147 assigned to the control group. EPVL was effective in facilitating the expulsion of stone fragments. The treatment group’s stone expulsion rate on the first day following EPVL was significantly greater than the control group’s (66.4% vs. 51.7%, *p* = 0.009). Stone clearance rates in the treatment and control groups were 63.2 and 55.1% at 1 week (*p* = 0.041), 84.9 and 70.7% at 2 weeks (*p* = 0.011), and 90.8 and 79.6% at 4 weeks (*p* = 0.017), respectively. The complications (hematuria, lumbago, and fever) between the groups did not show any significance (*p* > 0.05). Patients in the treatment group received an average of 5.2 sessions.

**Conclusion:**

EPVL is an efficient and secure procedure that facilitates lower pole stone discharge in obese patients following ESWL treatment. To support the aforementioned conclusions, additional large-scale multi-center prospective studies are required.

## Introduction

The World Health Organization defines obesity as having a body mass index (BMI) greater than 30 kg/m^2^ (1). As the economy rises, the prevalence of obesity rises annually and adversely affects human health (2). Obesity has been linked to hyperoxaluria and hypercalciuria in some studies, which are significant risk factors for the development of urinary stones (3, 4). The high incidence and recurrence rate of urolithiasis has also made it a global health issue (5). In the past 40 years, kidney stones have become more common among them. According to estimates, kidney stones will arise in 13% of men and 7% of women (6). Obese patients suffer urinary stones at a rate that is significantly greater than that of the general population (7). As a result, we should focus more on treating kidney stones in obese patients.

Due to its low invasiveness, ESWL is recommended as the first-line treatment for kidney stones under 2 cm in size (8, 9). However, residual stones after ESWL can lead to several issues, including urinary infection, renal colic, and the promotion of stone recurrence (10). Medical expulsive therapy and EPVL are two supplementary treatments for residual stones that can assist in stone-free rates (SFR) rise (11, 12). The EPVL is a brand-new device that promotes fragmentation by utilizing both gravity and mechanical vibrational forces (13).

To the best of our knowledge, EPVL has only sometimes been documented in the literature as a therapy for lower pole stones in obese patients. To assess the effectiveness and safety of EPVL for the treatment of lower pole stones following ESWL, we created this prospective randomized clinical research.

## Materials and methods

### Study design

**Figure 1:**
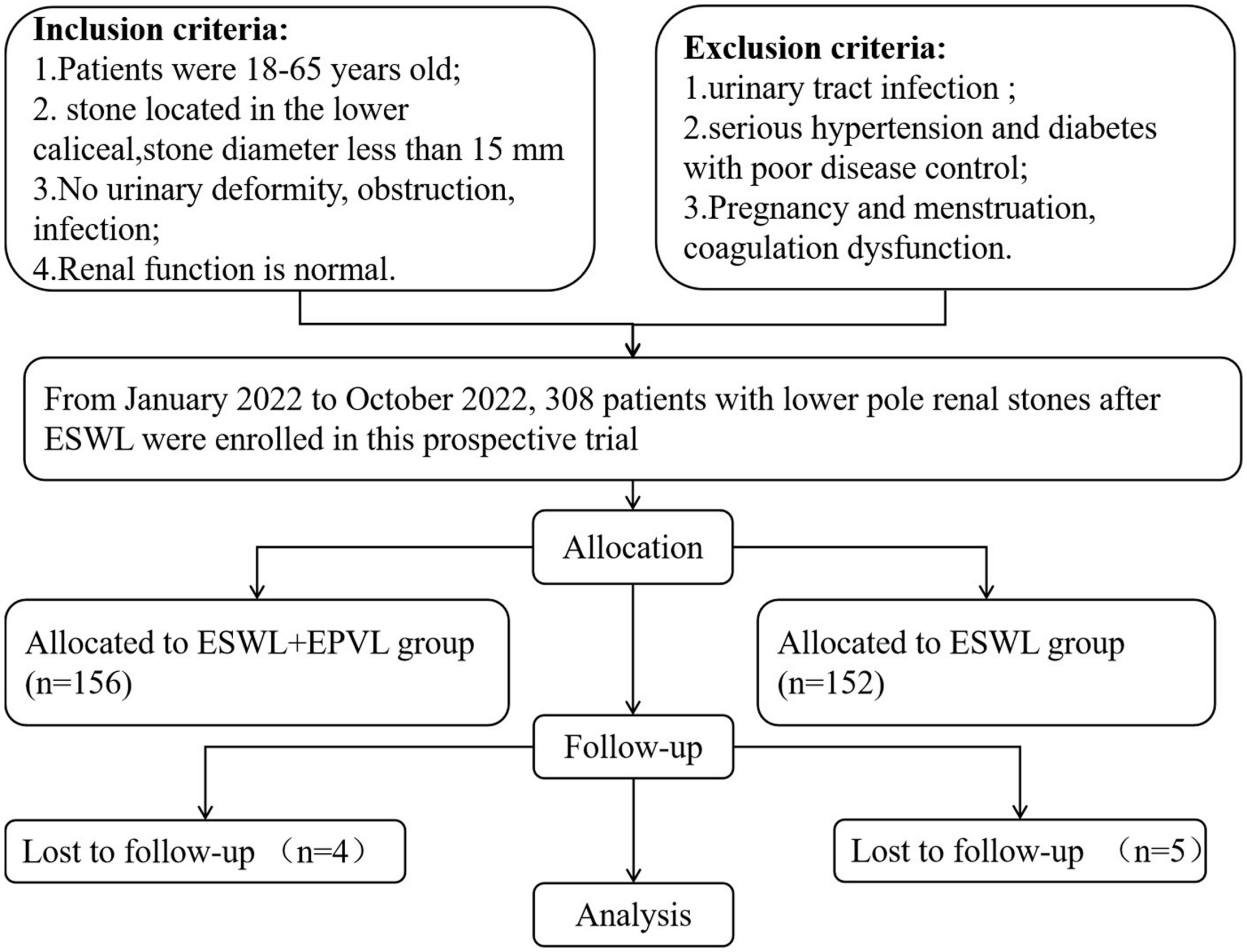
Flowchart for case selection.

The study is a prospective, controlled, open label, single-center investigation conducted from January to October 2022. The Chinese Clinical Trial Registry has accepted the protocol (ethics approval number: ChiCTR2200056402). This study enrolled 299 patients with lower pole stones sent to our institution (Figure 1). The preoperative evaluation included age, BMI, gender composition, chronic medical history, stone diameter, stone site, and ESWL history. All patients were informed about it, and their written informed consent was acquired.

### Randomization

After patients completed informed consent forms, research workers at our center used a computer-generated random number coding table to allocate eligible patients. The sealed envelope held the random assignment. Subjects were randomized in a 1:1 ratio, to receive EPVL or placebo for 4 weeks.

### Inclusion and exclusion criteria

Inclusion criteria: (1) patients ranged in age from 18 to 65 and had BMI greater than 30 kg/m^2^; (2) a stone in the lower pole had a diameter of <15 mm; (3) no obstruction, infection, or urinary deformity; (4) normal renal function. Exclusion criteria: (1) urinary tract infection; (2) severe hypertension and poorly controlled diabetes; (3) pregnancy and menstruation, coagulation dysfunction.

### Device mechanism

**Figure 2:**
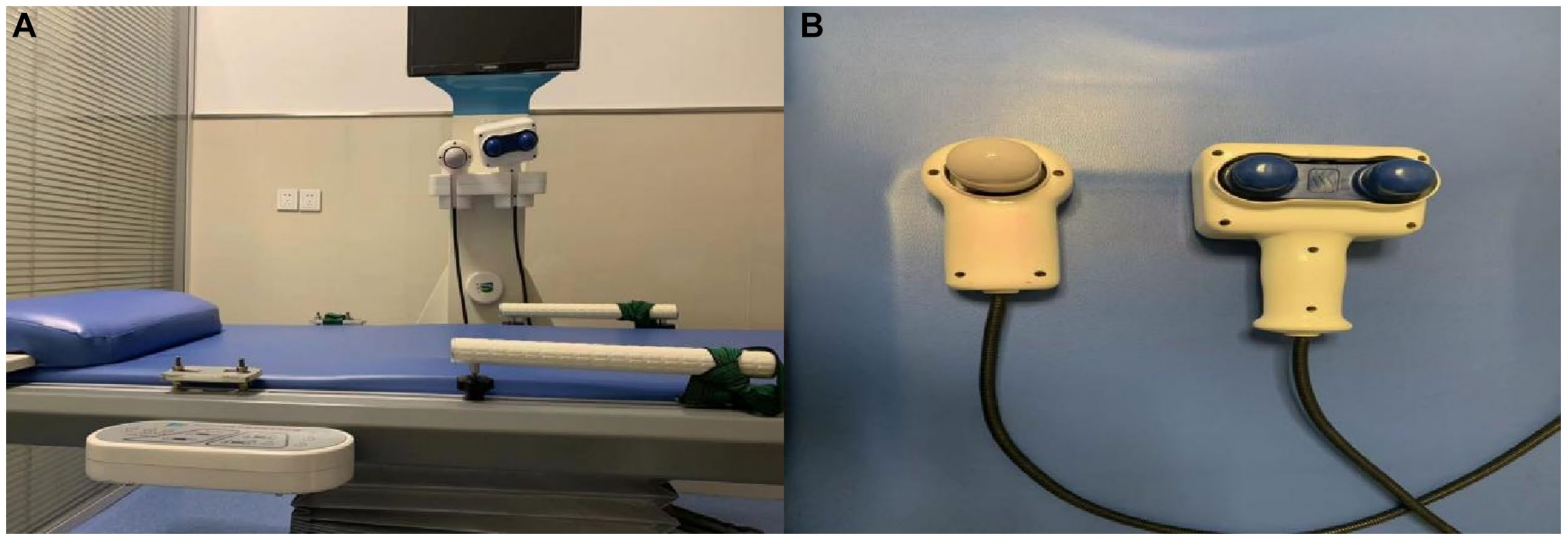
EPVL consists of a treatment bed **(A)** and oscillator **(B)**.

EPVL (Friend I, Fu Jian Da Medical Instrument Co., Ltd., Zhengzhou, China) is a new device to assist in stone removal. It comprises a treatment bed (with a rotating couch that has an angle of around 26 degrees) and two oscillators (Figure 2). The main oscillator is hand-held, while the sub-oscillator is put on the treatment bed. A multi-directional harmonic vibration approach is utilized by the device. During machine operation, the sub-oscillator vibrates harmonically in horizontal mode, causing lateral acceleration (vibration frequency: 1300–1900 blows/min; power: 200 W; amplitude: 5 mm), resulting in stone separation by vibration. Simultaneously, the axial force produced by the main oscillator was used to drive the stones out of the kidney (vibration frequency: 2800–3,500 blows/min; power: 40 W; amplitude: 5 mm). The stone is finally removed from the ureter while being adjusted in position and direction by the EPVL. Ultrasound was used during the procedure to track the changing stone position in real time.

### Study procedure

To demonstrate the existence of stone fragments after ESWL (Dornier Medical Systems, Weßling, Germany), all patients underwent non-contrasted computer tomography (NCCT). Patients received a single ESWL treatment, and the energy was controlled within 12–14 k V, the frequency was controlled within 60–90 times /min, and the number of shock was controlled within 2000–2,500 blows. All patients were reviewed at 1, 2, and 4 weeks after ESWL using plain abdominal X-ray for kidney-uretero-cystography and abdominal ultrasound, and NCCT scans were performed if necessary.

In the treatment group, 152 patients underwent EPVL without anesthesia after extracorporeal shock wave lithotripsy (ESWL). The patients were then placed in a supine position with their dorsal elevated and the main oscillator applied pressure to the ipsilateral renal calyx region after drinking approximately 1,500–2000 mL. The main oscillator frequency was gradually raised during this procedure by the patient’s tolerance (low: vibration frequency of 2,800 blows/min; High: vibration frequency of 3,500 blows/min). Each EPVL lasts 12–18 min, allowing the stone to pass from the lower renal calyx into the ureter. Ultrasound can be utilized intraoperatively to track the location of the stone and direct the tilt angle of the treatment bed to help with stone fragment removal. Usually three to four treatments can be performed per week, 1 day apart from each treatment.

In the control group, 147 patients were encouraged to drink more than 2000 mL/day and collect the fragments through a filter after ESWL. All of the collected fragments were analyzed.

### Follow up

The same follow-up was given to each patient, and no medication for stone removal was used. SFR is defined as the absence of residual stone or residual stone <2 mm in diameter (14).

NCCT noted the fragments of stone. Patients had EPVL treatment for one to six sessions. Failure of the treatment was determined to occur when stone fragments persisted after six sessions. Both the SFR and EPVL-related problems were noted. It was advised to resume ESWL treatment for patients who had no stone discharge at the end of the follow-up visit.

### Study outcomes

The first day’s stone expulsion rate and the SFR at weeks 1, 2, and 4 were the primary results. The complication associated with EPVL was a secondary outcome.

### Sample size and statistical analysis

Sample size calculation was determined by the pre-experimental results and our clinical experience. The SFR in the treatment group and control group were expected to be 87 and 73%, respectively. With a two-tailed of 0.05 and a (1 − β) of 0.80, we calculated the sample size by power analysis. This yielded a total sample of 275 patients, after considering a 10% dropout or missed follow-up patients. Finally, we expanded the sample size to 300 to compare the differences between the groups.

SPSS v.22.0 software for Windows (IBM Corp., Armonk, NY, United States) was used for statistical analysis. Categorical variables were analyzed using the Chi-square test and continuous variables were analyzed using the Student’s test. In all tests, *p* < 0.05 was considered to indicate a significant difference.

## Results

In this trial, 299 patients were randomly assigned to one of two groups: the treatment group (*n* = 152) or the control group (*n* = 147). Table 1 displays the demographic and clinical characteristics of the patients. The mean age of the treatment group was 41.7 ± 6.4 years versus 40.1 ± 8.1 years in the control group (*p* = 0.836). All patients received one ESWL session, whereas patients in the treatment group receive an average of 5.2 EPVL sessions. Age, BMI, gender, hypertension, diabetes history, mean stone size, stone position, Hounsfield units, and ESWL history were not significantly different between the two groups (all *p* > 0.05).

**Table 1:** Demographic and clinical characteristics.

Variables, mean ± SD or *n* (%)	ESWL+EPVL (*n* = 152)	ESWL (*n* = 147)	*p*
Age (year)	41.7 ± 6.4	40.1 ± 8.1	0.836
BMI (kg/m^2^)	33.49 ± 1.85	32.53 ± 1.69	0.213
**Gender**			
Male	109 (71.7)	105 (71.4)	-
Female	42 (28.3)	42 (28.6)	0.992
**Hypertension history**			
No	118 (77.6)	115(78.2)	-
Yes	34 (22.4)	32 (21.8)	0.817
**Diabetes history**			
No	134 (88.2)	127 (86.4)	-
Yes	18 (11.8)	20 (13.6)	0.759
Mean stone size(mm)	10.5 ± 0.2	10.2 ± 0.3	0.563
**Stone position**			
Left kidney	83 (54.6)	76 (51.7)	-
Right kidney	69 (45.4)	71 (48.3)	0.689
Hounsfield units	782.3 ± 123.7	769.3 ± 138.2	0.318
**ESWL history**			
No	115 (75.7)	113 (76.8)	-
Yes	37 (24.3)	34 (23.2)	0.735

*p* < 0.05 is considered statistically significant. BMI, body mass index; SD, standard deviation; ESWL, extracorporeal shock wave lithotripsy; EPVL, external physical vibration lithecbole.

Table 2 shows the differences in the clinical outcomes of patients. The rate of stone expulsion on the first day was considerably higher in the treatment group than in the control group (66.4% vs. 51.7%, *p* = 0.009). SFR was significantly higher in the treatment group than that in the control group at week 1, week 2 and week 4. However, there was no significant difference between the groups in terms of complications (hematuria, lumbago, and fever; *p* > 0.05). Stone composition in treatment and control group consisted of uric acid (39.8 vs. 38.2%), calcium oxalate (47.5 vs. 48.3%), and carboapatite (12.7 vs. 13.5%), respectively (all *p* > 0.05). At the end of the trial, a total of 24 patients in both groups had failed treatment, 14 in the treatment group and 10 in the control group, and all of these patients were recommended for surgery.

**Table 2:** Clinical outcomes and complications.

Variables, mean ± SD or *n* (%)	ESWL+EPVL (*n* = 152)	ESWL (*n* = 147)	*p*
Stone expulsion status (day 1)	101 (66.4)	76 (51.7)	0.009
SFR at the 1st weekend	96 (63.2)	81 (55.1)	0.041
SFR at the 2nd weekend	129 (84.9)	104 (70.7)	0.011
SFR at the 4th weekend	138 (90.8)	117 (79.6)	0.017
**Complications**			
Hematuria	17 (11.2)	24 (16.3)	0.157
Lumbago	8 (5.3)	18 (12.2)	0.312
Fever	3 (1.9)	6 (4.1)	0.594
Mean EPVL times	5.2 ± 0.8	–	–
**Stone analysis**			
Uric acid	39.8% (47/118)	38.2% (34/89)	0.86
Calcium oxalate	47.5% (56/118)	48.3% (43/89)	0.83
Carboapatite	12.7% (15/118)	13.5% (12/89)	0.81

*p* < 0.05 is considered statistically significant. SD, standard deviation; SFR, stone-free rates.

## Discussion

The treatment of kidney stones has undergone a significant evolution due to the constant advancement of medical technology, ranging from open surgery to ESWL, percutaneous nephrolithotomy (PCNL), and flexible ureteroscopy (FURS) (15). Although the SFR was improved by using these procedures, the residual stone is still a very upsetting issue (16). Repetition of renal colic, urinary tract infection, and stone recurrence are just a few of the complications that residual stones can cause. Due to the anatomy of the lower pole, such as the infundibular-pelvic angle and gravity-dependent position, it is particularly challenging to deal with residual stones at the lower pole, even when the stones have been completely crushed (17). In severe cases, this can even result in urosepsis, which can endanger patients’ lives. For these reasons, effective and timely resolution of residual stones is essential, not only to reduce the number of re-visits but also to enhance patients’ quality of life.

Obese patients with kidney stones are significantly more challenging to treat than normal-weight patients (18, 19). Due to the difficulty of localization and the distance between the skin and the kidney stone, ESWL is not as effective as RIRS or PCNL in treating obese patients (20). However, it does not necessitate hospitalization and generally results in a quick return to normal life (21). Moreover, both the risk of anesthesia and the financial burden is increased for obese patients (22). Consequently, ESWL patients were more likely to choose the same operation again.

Continual treatment and management of residual stones are required to further improve the quality of life of patients. EPVL is a technological evolution for treating residual stones following ESWL. In recent years, EPVL has been introduced, which combines mechanical vibration and gravitational effects to facilitate the passage of stone fragments, effectively increasing the SFR (23, 24). We reviewed the literature on the treatment of residual stones in the lower pole. Recently, Long et al. reported that EPVL is a viable treatment option for renal stones in the lower pole (25). Wu et al. conducted a randomized controlled trial to assess the effect of EPVL on upper urinary stones following ESWL. In his study, the SFR in the treatment group was significantly greater than in the control group for lower pole stones, although the difference was not statistically significant (*p* > 0.05) (26). Even though both studies assessed the efficacy of EPVL in patients with lower pole stones, the results are inconclusive, most likely due to the small sample sizes. In addition, the participants were primarily of normal weight, thus it is necessary to investigate the efficacy of this procedure in obese patients. In our study, the SFR in the treatment group was significantly higher than in the control group for the first, second, and fourth weeks (63.2% vs. 55.1%, 84.9 vs. 70.7%, and 90.8 vs. 79.2%, respectively). In terms of complications, we also discovered that EPVL did not raise the risk of lumbago, hematuria, or fever. It demonstrates that EPVL is a safe and effective supplementary treatment for residual stones in the lower pole following ESWL.

Our study indicates EPVL’s advantages for obese patients. First, our treatment offers a low risk of bleeding and a low financial load. Second, patients are not required to be hospitalized, they can return to their usual work and life sooner, and they are more receptive to treatment.

Nevertheless, this study has several limitations. First, all of the patients in this study have primary or secondary obesity, but not morbid obesity (BMI > 40 kg/m^2^). Second, the study is a single-center study with a small sample size, which may potentially generate a certain sampling error.

## Conclusion

Our study demonstrates that the combination of EPVL and ESWL is a safe and feasible therapeutic strategy for obese patients with lower pole stones (<15 mm). It offers obese people with kidney stones more therapy alternatives.

## Data availability statement

The original contributions presented in the study are included in the article/supplementary material, further inquiries can be directed to the corresponding author.

## Ethics statement

The studies involving humans were approved by the Chinese Clinical Trial Registry. The studies were conducted in accordance with the local legislation and institutional requirements. The participants provided their written informed consent to participate in this study. Written informed consent was obtained from the individual(s) for the publication of any potentially identifiable images or data included in this article.

## Author contributions

YL and JL designed the experiments. YL contributed to clinical data collection and assessment. YL analyzed the results. YL and JL wrote the manuscript. All authors contributed to the article and approved the submitted version.

## Conflict of interest

The authors declare that the research was conducted in the absence of any commercial or financial relationships that could be construed as a potential conflict of interest.

## Publisher’s note

All claims expressed in this article are solely those of the authors and do not necessarily represent those of their affiliated organizations, or those of the publisher, the editors and the reviewers. Any product that may be evaluated in this article, or claim that may be made by its manufacturer, is not guaranteed or endorsed by the publisher.
